# Chromodomain Helicase DNA-Binding Protein 5 Inhibits Renal Cell Carcinoma Tumorigenesis by Activation of the p53 and RB Pathways

**DOI:** 10.1155/2020/5425612

**Published:** 2020-09-28

**Authors:** Sheng Huang, Qitao Yan, Shilin Xiong, Yiqi Peng, Rui Zhao, Chunxiao Liu

**Affiliations:** ^1^Department of Urology, ZhuJiang Hospital of Southern Medical University, Guangzhou 510282, China; ^2^Guangdong Provincial Key Laboratory of Geriatric Infection and Organ Function Support, Department of Medical Intensive Care Unit, General Hospital of Southern Theatre Command, Guangzhou 510010, China; ^3^Department of Biochemistry and Molecular Biology, School of Basic Medical Sciences, Southern Medical University, Guangzhou 510515, China

## Abstract

Chromodomain helicase DNA-binding protein 5 (CHD5) plays a crucial tumor suppressor role in multiple types of tumors. For this study, we investigated its clinical significance and the molecular mechanism(s) underlying tumorigenesis in renal cell carcinoma (RCC). Initially, CHD5 expression was assessed in primary tumor tissue and in tissue array. Correlations among CHD5 expression and clinicopathological characteristics were analyzed. Next, lentivirus-mediated CHD5 overexpression in the ACHN and 769-P cells was used to assess effects on proliferation, migration, invasion ability, and the regulation of the p14^ARF^/p53 and p16^INK4a^/RB signaling pathways. Finally, a xenograft mouse model was used to verify its impact on tumor growth *in vivo*. Results demonstrated that CHD5 was downregulated in tumor tissues and that low CHD5 expression was correlated with advanced TNM stage, high Fuhrman grade, lymph node metastasis, and poor survival. Overexpression of CHD5 inhibited proliferation, migration, and invasion *in vitro*; prompted cell cycle G1 phase arrest; induced apoptosis; and suppressed tumor growth *in vivo*. Furthermore, we confirmed that CHD5 activates the p53 and RB pathways to inhibit tumorigenesis in RCC. In summary, CHD5 is involved in the initiation and progression of RCC and may serve as a diagnostic biomarker and a potential therapeutic target for RCC.

## 1. Introduction

Renal cell carcinoma (RCC) is the third most common tumor in the genitourinary system, representing 3% of all cancers worldwide, and the incidence has increased annually by 2% during the past decades [1, 2]. However, RCC is the most lethal genitourinary cancer due to its high recurrence rate, metastasis rate, and resistance to radiotherapy and chemotherapy [3]. Therefore, discovering new tumor biomarkers and exploring the molecular mechanism(s) involved in the tumorigenesis of RCC would be beneficial for developing new diagnostic and therapeutic strategies.

Human *1p36* has been reported to be deleted in multiple types of tumors, including those of neurogenic, epithelial, and hematopoietic origins [4]. Bagchi et al. identified CHD5 as a tumor suppressor gene (TSG) mapped to *1p36* by a chromosome engineering approach in a mouse model and confirmed that CHD5 plays a tumor suppressor role in human neuroblastoma [5]. Subsequent studies demonstrated that CHD5 is downregulated in neuroblastoma and is correlated with unfavorable clinical features and poor survival; restoration of CHD5 expression could inhibit clonogenicity and tumorigenicity [6, 7]. Evidence soon emerged that CHD5 is downregulated by promoter hypermethylation and/or deletion in many other types of tumors, such as gastric cancer [8], colorectal cancer [9, 10], prostate cancer [11], laryngeal squamous cell carcinoma [12], lung cancer [13, 14], hepatocellular carcinoma [15], ovarian cancer [16], breast cancer [17], leukemia [18], pancreatic cancer [19], and glioma [20]. Moreover, low expression of CHD5 correlated with unfavorable clinical features and poor survival [21]. Therefore, downregulation of CHD5 may be a critical initiating molecular event in tumorigenesis and represent a prognostic biomarker of outcome in patients with cancer. However, only one previous study has reported that CHD5 is inactivated by promoter methylation in RCC [22]. The clinical significance of CHD5 expression in RCC and the molecular mechanism underlying tumorigenesis remain unclear.

In this study, we detected the expression of CHD5 in RCC tissues and cell lines and further assessed the correlation between CHD5 expression and clinicopathological characteristics. Subsequently, we used a gain-of-function assay to evaluate its effects on cell proliferation, migration, and invasion, as well as the key signaling pathways involved in the regulation of CHD5. Our study showed that downregulation of CHD5 expression was related to advanced TNM stage, high Fuhrman grade, lymph node metastasis, and short overall survival. Overexpression of CHD5 induced G1 phase arrest and apoptosis, thereby inhibiting proliferation *in vivo* and *in vitro*, and suppressed migration and invasion *in vitro*. CHD5 positively regulated the p14^ARF^/p53 and p16^INK4a^/RB pathways to exert tumor suppressor functions. In conclusion, we elucidated the role of CHD5 in the tumorigenesis of RCC, which might provide a new insight for the diagnosis and treatment of renal cancer.

## 2. Materials and Methods

### 2.1. Clinical Tissues and Cell Lines

Twenty-four pairs of fresh primary tumor tissues and adjacent tissues were obtained from ZhuJiang Hospital of Southern Medical University. Clinicopathological information was collected from hospital records. Informed consent was obtained from all patients with approval of the Ethics Committee of ZhuJiang Hospital of Southern Medical University.

Human RCC cell lines (786-0, 769-P, Caki-1, ACHN, and A498) and normal epithelial cell lines (HEK293 and HK-2) were ordered from the Cell Bank of the Chinese Academy of Sciences (Shanghai, China). RCC cell lines were grown in RPMI-1640 medium (Gibco, Grand Island, NY, USA) with 10% fetal bovine serum (FBS; Gibco). HEK293 and HK-2 were grown in Dulbecco's modified Eagle's medium (DMEM; Gibco) and DMEM/F12 (Gibco) with 10% FBS, respectively. All cells were incubated at 37°C with 5% CO_2_ atmosphere.

### 2.2. Immunohistochemistry Assay

The tissue array (Outdo Biotech, Shanghai, China) was applied for immunohistochemistry (IHC) staining to detect CHD5 expression in 90 RCC patients. Antigen unmasking was performed in citrate buffer by microwaving after deparaffinization and rehydration, followed by inactivation of endogenous peroxidase with 0.3% H_2_O_2_. Sections were blocked with goat serum (ZSGB-BIO, Beijing, China) and incubated with CHD5 (Cell Signaling Technology, Beverly, USA; 1 : 100 dilution) overnight at 4°C. Subsequently, sections were probed with anti-rabbit antibody and avidin-biotin peroxidase at room temperature and visualized using diaminobenzidine before counterstaining with hematoxylin. The percentage of positive cells (PP) were graded as follows: 0 (<1%), 1 (1-10%), 2 (11-50%), and 3 (>50%). Staining intensity (SI) was defined as follows: no staining, weak staining, moderate staining, and strong staining, corresponding to 0-3 points. PP multiplied by SI was identified as immunoreactivity scoring (IRS). IRS 0–1 was defined as low expression, and >1 was defined as high expression [19].

### 2.3. Lentivirus-Mediated Overexpression of CHD5

To generate CHD5 overexpression (CHD5-OE) cells using CRISPR/Cas9 gene editing technology, dCas9 vectors and sgRNA targeting the CHD5 gene were cloned into dCas-VP64-Puro and sgRNA-MS2-P65-HSF1-Neo, respectively. The LV-dCas9 and LV-sgRNA lentivirus were produced by GeneChem (Shanghai, China). We designed three pairs of sgRNA oligonucleotides, and the sequences were as follows: sgRNA1 (“CCTCGGCCGGCTGCGGGACT”), sgRNA2 (“CGGCGGCAGCGCCAGAGGCA”), and sgRNA3 (“GCCCGGGCTTTGCGGGGAGC”). The LV-dCas9-VP64 lentivirus was seeded in the ACHN and 769-P cells at a multiplicity of infection (MOI) of 20 and screened with puromycin (Beyotime, Shanghai, China) at a final concentration of 2 *μ*g/ml for 5–7 days. Subsequently, the LV-sgRNA lentivirus was seeded and the empty vector served as the control. The CHD5 overexpression cells, empty vector transfected cells, and untransfected cells were harvested 7–10 days later, which were defined as CHD5-OE, Ctrl-OE, and NC cells, respectively.

### 2.4. RNA Isolation and Quantitative Reverse Transcription PCR (RT-qPCR)

RNA was obtained by dissolving tissues and cell lines using TRIzol (Invitrogen, Carlsbad, CA, USA), then reverse transcribed into cDNA using a PrimeScript RT Reagent Kit with gDNA Eraser (TaKaRa, Japan), and the expression of CHD5 was detected by SYBR Premix Ex Taq II (TaKaRa, Japan). Primers sequences were as follows: CHD5: 5′-CGAAGGCTACAAGTATGAGCGG-3′ and 5′-GGTTGAGAGGAGGAAGCAGAAC-3′; MMP-9: 5′-CTGGAGACCTGAGAACCAATC-3′ and 5′-CAGAGATTTCGACTCTCCACG-3′; MMP-2: 5′-GATAACTCTGGACTTAGACCGC-3′ and 5′-CAGCCATAGAAGGTGTTCAGG-3′; and *β*-actin: 5′-CATGTACGTTGCTATCCAGGC-3′ and 5′-CTCCTTAATGTCACGCACGAT-3′. The relative expression of CHD5 was analyzed by the 2^-*ΔΔ*T^ method and normalized with *β*-actin.

### 2.5. Western Blot

Proteins were isolated from cell lines and tissues by the RIPA lysate (Beyotime), then separated by electrophoresis on 8–15% SDS-polyacrylamide gel and transferred to PVDF membrane (Bio-Rad). After blockage with 5% skim milk for 1 hour, the primary antibody (Cell Signaling Technology) was incubated at 4°C overnight and then probed with a secondary antibody (Beyotime). Signals were visualized by ECL Western Blotting Detection Reagents (Epizyme, Shanghai, China).

### 2.6. Cell Counting Kit-8 (CCK-8) Analysis

Cell proliferation was assessed by CCK-8 assay. 2 × 10^3^cells were inoculated into 96-well plates and incubated for cell attachment. Ten microliters of the enhanced CCK-8 reagent (Beyotime) was added and incubated for 2 hours; then, the absorbance was measured at 450 nm with Infinite M200 (Tecan, Switzerland). Thereafter, measurements were taken every 24 hours until 96 hours.

### 2.7. Plate Colony Formation Assays

The 769-P and ACHN (500 cells per dish) cells were seeded onto 35 mm dishes. After two weeks of culture, colonies were stained with 0.1% crystal violet (Beyotime) and counted with a light microscope.

### 2.8. Flow Cytometry Analysis

To examine apoptotic cells, cells from each group were harvested and resuspended in 195 *μ*l of binding solution. Then, 5 *μ*l of Annexin V-FITC and 10 *μ*l of propidium iodide staining solution were added and mixed gently. After incubation at room temperature in the dark for 15 minutes, the apoptotic cells were detected by flow cytometry (BD Biosciences, MA, USA).

For cell cycle analysis, cells were fixed with ice-cold 70% ethanol for 12 hours; then, propidium iodide staining solution and RNase A (Beyotime) were added and bathed for 30 minutes at 37°C. Red fluorescence was detected by flow cytometry (BD Biosciences), and DNA content was analyzed using ModFit LT software.

### 2.9. Wound Healing and Transwell Assays

Wound healing assay was carried out to assess migration ability. The 769-P and ACHN cells were seeded into 6-well plates and maintained until cell confluence reached 90–100%, scratched with 10 *μ*l micropipette tips, and then cultured in serum-free medium. Images were captured with an inverted microscope at 0 and 24 hours after the scratch.

Transwell assay was performed in a 24-well transwell chamber (Corning, NY, USA) to evaluate cell invasion capacity. Matrigel (BD Biosciences) was diluted to 300 *μ*g/ml to coat the chamber. Subsequently, the upper and low chambers were supplied with 100 *μ*l of serum-free medium containing 10^5^ cells and 600 *μ*l medium with 10% FBS, respectively. Following 24 hours of incubation, the cells on the surface of the membrane were gently wiped with a cotton swab, and the cells invading into the membranes were stained with 0.1% crystal violet. Five fields were randomly observed with a microscope.

### 2.10. Xenograft Mouse Tumor Models

Eighteen 4-5-week-old BALB/c nude mice were obtained from the Experimental Animal Center of Southern Medical University. Mice were randomized into three groups (NC, Ctrl-OE, and CHD5-OE) with six mice per group. The ACHN cell suspensions were mixed with an equal volume of Matrigel (BD Biosciences), and then 5 × 10^6^ cells were administered into the right axilla of mice. The length (*L*) and width (*W*) of the tumors were recorded once a week, and tumor volumes ((*L* × *W*^2^)/2) were calculated. On day 28, mice were sacrificed and tumors were resected. The experiments were approved by the Animal Research Ethics Committee of Southern Medical University.

### 2.11. Statistical Analysis

SPSS software version 20.0 (SPSS Inc., Chicago, IL, USA) was used to analyze the data, and the data were presented as mean ± standard error of the mean (SEM). *t*-test or Mann-Whitney *U* test were conducted to analyze differences between the two groups. One-way ANOVA was performed to compare the differences among multiple groups, and Kaplan-Meier survival analysis was used to compare patients' overall survival time. *p* < 0.05 was defined as statistically significant.

## 3. Results

### 3.1. CHD5 Is Downregulated and Correlated with Adverse Clinicopathological Characteristics in RCC Patients

**Figure 1:**
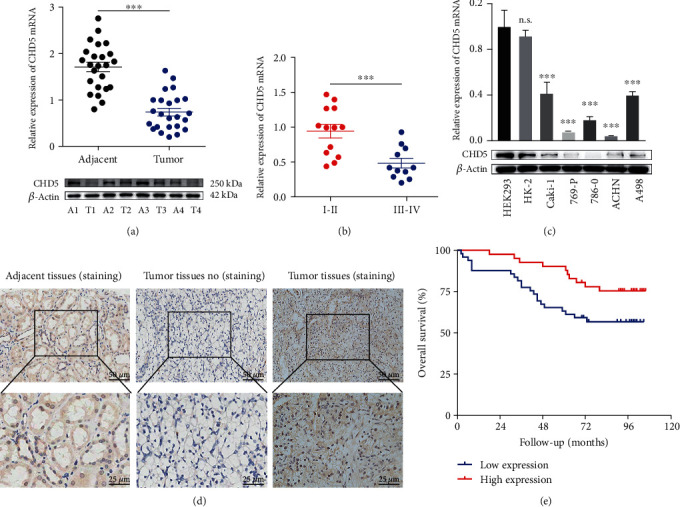
Expression of CHD5 in RCC tissues and cell lines. (a) CHD5 was downregulated in 24 RCC tumor tissues (T) compared to adjacent tissues (A). (b) CHD5 expression in TNM stage III-IV was lower than that in stage I-II. (c) CHD5 is downregulated in RCC cell lines Caki-1, 769-P, 786-0, ACHN, and A498, compared to HEK293 and HK-2. (d) Representative IHC staining with CHD5 in tumor and adjacent tissues. (e) Kaplan-Meier survival analysis assessed the correlation between CHD5 expression and overall survival. ^∗∗∗^*p* < 0.001. n.s.: no significance.

To understand the role of CHD5 in RCC, we first measured the mRNA and protein levels in 24 paired tumor and adjacent tissues. The results showed that the expression of CHD5 was obviously downregulated in tumor tissues compared to matched adjacent tissues (Figure 1(a)). In advanced pathological stages (stages III and IV), CHD5 levels were lower than those in primary pathological stages (stages I and II) (Figure 1(b)). Similarly, downregulation of CHD5 was also observed in five human RCC cell lines compared to normal renal epithelial cells HK-2 and HEK293 (Figure 1(c)). Subsequently, the correlation between CHD5 expression levels and clinicopathological characteristics was assessed and the results showed that low CHD5 expression was closely related to advanced TNM stage, high Fuhrman grade, and lymph node metastasis; nevertheless, there was no relationship to patients' gender, age, and tumor size (Table 1). In addition, Kaplan-Meier survival analysis of tissue array immunohistochemical staining results showed that patients with low CHD5 expression had a shorter overall survival than patients with high CHD5 expression (Figures 1(d) and 1(e)). Taken together, these data indicate that CHD5 is involved in the tumorigenesis and has a significant correlation with the prognosis of patients with RCC.

### 3.2. CHD5 Overexpressed in the ACHN and 769-P Cells by the CRISPR/dCas9 SAM System

**Figure 2:**
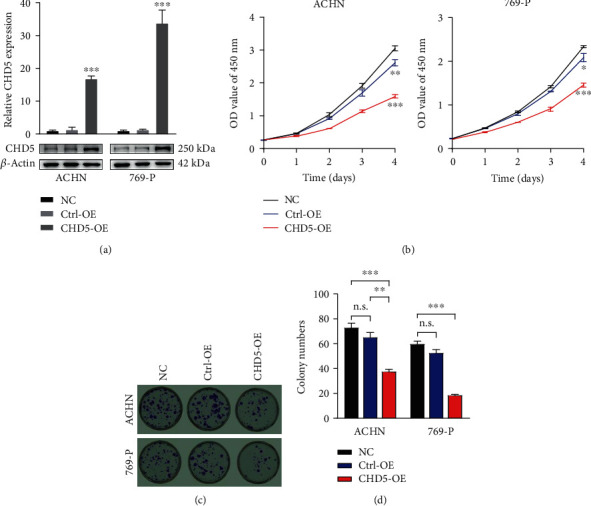
CHD5 inhibits cell proliferation *in vitro*. (a) CHD5 expression in the ACHN and 769-P cells transfected with lentivirus in comparison to the NC and Ctrl-OE groups. (b) CCK-8 assay examined the proliferation in the ACHN and 769-P cells. (c, d) Colony formation assays in the ACHN and 769-P cells. ^∗∗∗^*p* < 0.001, ^∗∗^*p* < 0.01, and ^∗^*p* < 0.05. n.s.: no significance.

The CRISPR/dCas9 SAM (synergistic activation mediator) system is a method for endogenously increasing the expression of target genes that was first proposed and established by Konermann and colleagues [23]. In brief, the dCas9-VP64 fusion protein (without endonuclease activity) binds to the target gene promoter region with the guidance of sgRNA to recruit transcription activation complex MS2-P65-HSF1, thereby increasing the expression of the target gene [23]. In the present study, we first designed three pairs of sgRNAs targeting the CHD5 gene on the CRISPR Design (http://crispr.mit.edu) and cloned them into the LV-sgRNA-MS2-P65-HSF1-Neo vector. Subsequently, the dCas9-VP64 fusion protein and sgRNA-MS2-P65-HSF1 sequence expression frame were introduced into the ACHN and 769-P cells by SAM double-vector lentivirus, respectively. RT-qPCR and western blot were performed to verify the transfected efficiency. The results indicated that the sgRNA3 sequence could significantly increase the expression of CHD5 in the ACHN and 769-P cells (Figure 2(a)).

### 3.3. CHD5 Overexpression Inhibits RCC Cell Proliferation *In Vitro*

To illustrate the biological role of CHD5 in RCC, the CCK-8 and clone formation assays were performed to determine cell proliferation *in vitro*. CCK-8 assay demonstrated that the proliferation rate of the ACHN and 769-P cells overexpressing CHD5 was significantly lower than that of the Ctrl-OE and NC groups (Figure 2(b)), and clone formation assays also verified the inhibitory effect of CHD5 on proliferation *in vitro* (Figures 2(c) and 2(d)).

### 3.4. CHD5 Prompts Cell Cycle G1 Phase Arrest and Induces Apoptosis

**Figure 3:**
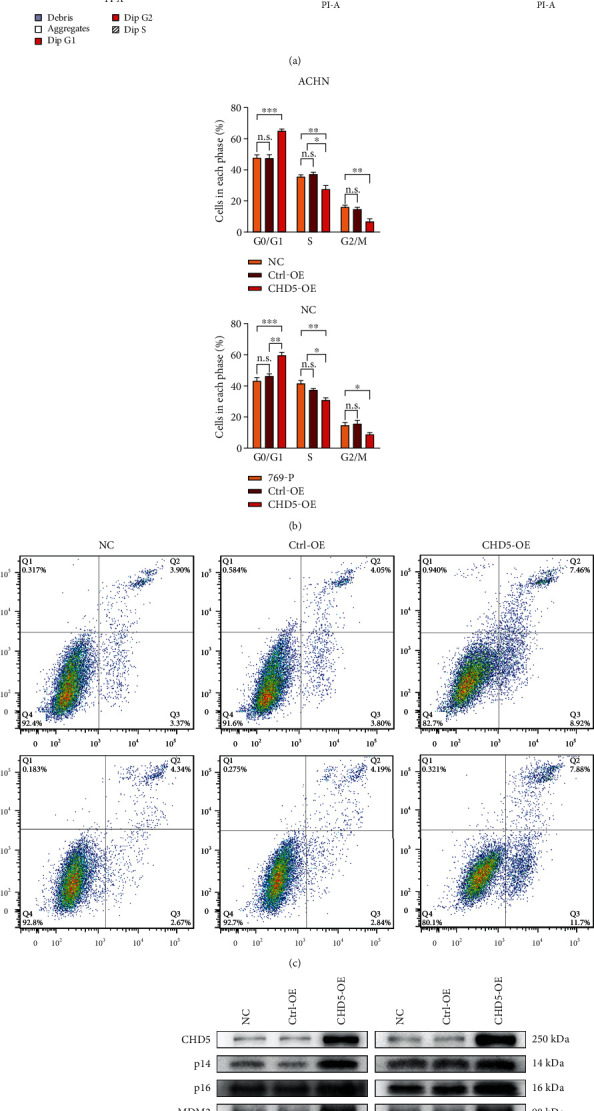
Effect of CHD5 on cell cycle and apoptosis. (a, b) Distribution of cell cycle in different phases analyzed by flow cytometry. (c, d) Percentage of apoptotic cells detected by flow cytometry. (e) The influence of CHD5 on the p14^ARF^/p53 and p16^INK4a^/RB pathways by western blot. ^∗∗∗^*p* < 0.001, ^∗∗^*p* < 0.01, and ^∗^*p* < 0.05. n.s.: no significance.

We used flow cytometry to evaluate the cell cycle and apoptosis, as these factors are closely related to cell proliferation. After overexpression of CHD5 in the ACHN and 769-P cells, the cell ratio in G1 phase increased notably, while that in the S and G2 phases decreased compared with the those in the Ctrl-OE and NC groups (Figures 3(a) and 3(b)). Apoptosis detection results showed that the number of apoptotic cells also increased significantly in both the ACHN and 769-P cells overexpressing CHD5 (Figures 3(c) and 3(d)). The results indicate that CHD5 induces cell cycle G1 phase arrest and apoptosis to inhibit proliferation.

### 3.5. CHD5 Activates the p16^INK4a^/p53 and p14^ARF^/RB Pathways

The INK4a/ARF locus encoded the tumor suppressor proteins, p16^INK4a^ and p14^ARF^ (p19^ARF^ in mice), prompting the activities of RB and p53 to regulate cell growth [24]. Studies have shown that the p53 and RB pathways are inactivated in a variety of tumors, leading to cell cycle disorder and disruption of apoptosis [25]. A previous study has shown that CHD5 positively regulated p19^ARF^/p53-mediated pathways in mouse models and loss of CHD5 function is prone to malignant transformation by impairing the p19^ARF^/p53 pathway [5]. However, it is unclear whether CHD5 has similar regulatory effects on INK4a/ARF in different species and cells. Therefore, we predicted that CHD5 mediates the involvement of INK4a/ARF in the regulation of biological behavior in RCC. We examined the key proteins of the p16^INK4a^/RB- and p14^ARF^/p53-mediated pathways using western blot. As shown in Figure 3(e), the expression of p14^ARF^, MDM2, p53, p21, Bax, caspase-9, p16^INK4a^, and RB increased, while the expression of p-RB, cyclin D1, and CDK4 decreased. This suggests that CHD5 regulates cell cycle and apoptosis by activating the p14^ARF^/p53 and p16^INK4a^/RB pathways.

### 3.6. CHD5 Suppresses Migration and Invasion

**Figure 4:**
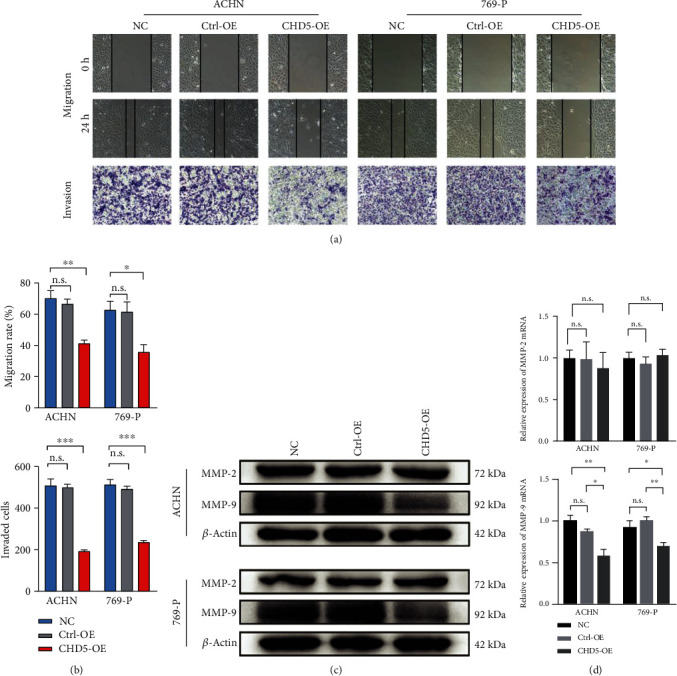
CHD5 suppresses migration and invasion of the ACHN and 769-P cells. (a, b) Migration and invasion ability were examined by wound healing and transwell assays. (c, d) MPP-2/9 expression levels were detected by western blot and RT-qPCR. ^∗∗∗^*p* < 0.001, ^∗∗^*p* < 0.01, and ^∗^*p* < 0.05. n.s.: no significance.

As shown in Table 1, CHD5 low expression is related to lymph node metastasis. Therefore, we next investigated the effect of CHD5 on the migration and invasion in RCC cell lines. Wound healing and transwell assays demonstrated that overexpression of CHD5 significantly suppressed the migration and invasion of the ACHN and 769-P cells (Figures 4(a) and 4(b)). We further explored the potential mechanism of CHD5 inhibition of cell motility. Western blot and RT-qPCR were performed to detect previously reported MMPs (MMP-2 and MMP-9), which were closely correlated with the progression of RCC [26]. The results showed that MMP-9 significantly decreased in cells overexpressing CHD5 (Figures 4(c) and 4(d)). Therefore, we conclude that CHD5 may inhibit RCC metastasis, at least in part, through the regulation of MMP-9 expression.

### 3.7. CHD5 Inhibits Tumor Growth *In Vivo*

**Figure 5:**
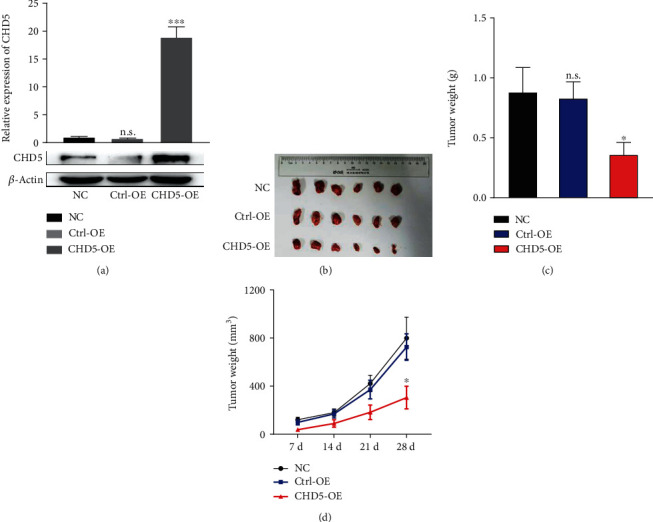
CHD5 inhibits tumor growth *in vivo*. (a) The efficiency of CHD5 overexpressing in ACHN verified by WB and RT-qPCR. (b) Tumors were resected after 4 weeks of ACHN cell injection. (c) Tumor weight was measured after 4 weeks. (d) Tumor size was recorded every week. ^∗∗∗^*p* < 0.001 and ^∗^*p* < 0.05. n.s.: no significance.

To further investigate the effect of CHD5 on tumorigenesis *in vivo*. ACHN cells with stable expression of CHD5 (Figure 5(a)) were injected subcutaneously into BALB/c nude mice. Four weeks later, mice were sacrificed and tumors were resected (Figure 5(b)). The tumor mass was weighed, and the tumor volume was calculated. It was found that the average tumor weight (Figure 5(c)) and average tumor volume (Figure 5(d)) of the CHD5-OE group were significantly lower than those of the Ctrl-OE and NC groups. Therefore, overexpression of CHD5 significantly inhibits tumorigenesis *in vivo*.

## 4. Discussion

RCC is the malignant tumor with the highest mortality rate in the urogenital system. Over the past decade, there has been some progress in the treatment of RCC, such as the advent of targeted agents and immune checkpoint inhibitors [27]; however, the clinical outcomes of patients with RCC are still unsatisfactory [28, 29]. Hence, it is necessary to identify new biomarkers that provide valuable information for clinicians to choose appropriate treatment options and predict prognosis of patients.

CHD5 is the fifth member of the chromatin remodeling family and is involved in regulating the structure and transcription of chromatin [21]. CHD5 binds to unmodified N-terminus of histone 3 for tumor suppression [30]. Increasing evidence shows that CHD5 plays a critical role in human cancer initiation and progression [21]. Studies on neuroblastoma have found that high CHD5 expression is closely related to favorable clinical and biologic characteristics, while low expression or deletion is related to adverse characteristics such as MYCN amplification and poor prognosis. Interestingly, induction chemotherapy could restore the expression of CHD5 in tumor tissues of half of high-risk neuroblastoma patients and present good response to chemotherapy and radiotherapy [7, 31]. The study by Du et al. revealed that CHD5 was downregulated in gallbladder cancer, and low expression of CHD5 was associated with poor clinical and pathological characteristics; and the patients with low expression of CHD5 had shorter overall survival and disease-free survival [32]. Similar results were observed in other tumors [19, 20, 33–35]. Herein, we reported the prognostic value of CHD5 expression in RCC patients. In brief, CHD5 is significantly downregulated in RCC tissues and cell lines, and downregulation of CHD5 was closely related to advanced TNM stage, high Fuhrman grade, and lymph node metastasis, as well as poor overall survival. Taken together, CHD5 was involved in the initiation and progression of RCC, and a decrease in CHD5 expression was associated with poor clinical prognosis.

Unlimited proliferation of tumor cells often involves cell cycle disorder and apoptotic response inactivation [36, 37]. The INK4a/ARF locus encoded two gene products, p16^INK4a^ and p14^ARF^ (p19^ARF^ in mice), which function by promoting the activity of RB and p53 transcription factors to regulate the cell cycle and apoptosis [24]. The disruption of p14^ARF^/p53 and p16^INK4a^/RB is involved in tumorigenesis of multiple tumors [38]. Moreover, the p16^INK4a^/p14^ARF^ axis has been previously reported to be involved in the development of RCC [39]. In addition, Bagchi et al. found that CHD5 could positively regulate INK4a/ARF in mice and CHD5 deficiency is prone to malignant transformation by impairing the p19^ARF^/p53 pathway [5]. Nevertheless, it is unclear whether it has a similar effect on p14^ARF^ in human RCC cells. In our present study, we revealed that CHD5 overexpression increased the expression level of p14^ARF^ and p16^INK4a^ and then activated the p53 and RB pathways, resulting in a decrease in cyclin D1 and CDK4 expression and an increase in Bax and caspase-9 expression, thereby prompting G1 phase arrest and inducing apoptosis. These results suggested that a potential mechanism underlying CHD5 inhibition of RCC proliferation might be dependent on the activation of the p53 and RB signaling pathways by the INK4a/ARF locus.

Distant metastasis is the leading cause of death in RCC patients. Degradation of the extracellular matrix (ECM) by matrix metalloproteinase (MMP) is a key process in tumor metastasis and progression. Studies have shown that MMPs are frequently upregulated in RCC, including MMP-2/7/9/14/17 [40, 41]. In particular, MMP-2/9 is strongly correlated with poor survival in clear cell RCC, as MMP-2/9 is also involved in angiogenesis, which has a crucial role in the progression of highly vascularized malignancies, such as RCC [26]. Therefore, MMP-2/9 expression is higher in patients with metastatic RCC, compared with localized RCC [42]. In addition, a study has shown that the introduction of wild-type p53 into p53 mutant human soft tissue sarcoma cells could reduce the expression of MMP-9 mRNA and protein and decrease the proteolytic activity of MMP-9 [43]. Our data showed that low expression of CHD5 in RCC was closely associated with advanced TNM stage and lymph node metastasis. Moreover, CHD5 overexpression in the ACHN and 769-P cells suppressed migration and invasion, upregulated p53, and decreased the expression of MMP-9. This suggested that CHD5 might be involved in RCC metastasis through downregulation of MMP-9 expression mediated by p53. Certainly, this needs to be confirmed by a series of follow-up studies.

## 5. Conclusions

Our study indicates that CHD5 is downregulated in RCC and is closely associated with adverse clinicopathological features and poor outcomes. CHD5 inhibits the tumorigenesis by activating the p14^ARF^/p53 and p16^INK4a^/RB pathways. These data suggest that CHD5 could be a diagnostic biomarker and potential therapeutic target for RCC.

## Figures and Tables

**Table 1:** Correlation between CHD5 expression levels and clinicopathological features in RCC patients.

Variables	Number (%)	mRNA^a^ (mean ± SEM)	*p* value	Protein^b^ (mean ± SEM)	*p* value
Gender					
Male	17 (70.8)	0.474 ± 0.061	0.573	0.629 ± 0.117	0.508
Female	7 (29.2)	0.409 ± 0.094		0.494 ± 0.125	
Age					
<60	14 (58.3)	0.532 ± 0.068	0.071	0.722 ± 0.135	0.101
≥60	10 (41.7)	0.348 ± 0.064		0.404 ± 0.079	
Tumor size					
<7	14 (58.3)	0.545 ± 0.072	0.069	0.738 ± 0.139	0.061
≥7	10 (41.7)	0.330 ± 0.047		0.382 ± 0.050	
TNM stage					
I-II	13 (54.2)	0.630 ± 0.054	<0.001	0.797 ± 0.138	0.008
III-IV	11 (45.8)	0.249 ± 0.029		0.344 ± 0.049	
Fuhrman grade					
I-II	12 (50.0)	0.603 ± 0.074	0.004	0.811 ± 0.153	0.057
III-IV	12 (50.0)	0.308 ± 0.035		0.368 ± 0.038	
Lymph node metastasis					
Yes	6 (25.0)	0.203 ± 0.014	0.002	0.307 ± 0.046	0.028
No	18 (75.0)	0.539 ± 0.054		0.684 ± 0.111	

^a^Relative expression of CHD5 ratio by RT-qPCR. ^**b**^Grayscale ratio by western blot.

## Data Availability

The data used to support the findings of this study are included within the article.
